# Preliminary Results of a Cognitive Training Program in a Memory Clinic

**DOI:** 10.1093/geroni/igaf122.2229

**Published:** 2025-12-31

**Authors:** Dev Ashish, Molly Maxfield

**Affiliations:** Banner Alzheimer’s Institute, Tucson, Arizona, United States; Arizona State University, Phoenix, Arizona, United States

## Abstract

**Background:**

Patients with mild cognitive impairment (MCI) or early stages of dementia often search for treatments to improve cognition and help manage deficits. Care partners also seek information to provide better care. Multi-component non-pharmacological interventions provided to dementia patients with caregivers have similar benefits as pharmacological treatments but without side effects. Here, we present information about the Multicomponent Comprehensive Intervention with Care-partners (MCI-Care) at Banner Alzheimer’s Institute in Tucson.

**Method:**

Twenty-one sessions for dyads (patients with MCI or early stages of dementia and their care-partners) include psychoeducation, training, and problem-solving for 1) healthy brain behaviors and 2) compensatory strategies, combined with 3) cognitive training for attention, processing speed, executive functioning, and memory domains. Sixteen dyads started the treatment. Brief pre- and post-assessments included a cognitive screen and self-report questionnaires. Dyads completed a brief exit survey about their experiences.

**Results:**

Eleven of 16 patients completed training. Patients demonstrated improvement in objective cognition and self-reported measures. Care-partners endorsed improvement in patients’ cognition, function, and their caregiver burden. All patients who completed the treatment found it informative and helpful; all indicated they were “very likely” to recommend this training. Three patients with MCI did not complete the training after their mood improved. Two patients with dementia discontinued, needing more support with caregiving resources.

**Conclusion:**

MCI-Care is best tolerated by patients without significant mood issues or need for caregiving resources. There is evidence for the acceptability and effectiveness of the intervention. Future research will establish statistical and clinical significance of dyadic and group interventions.

